# Comparison of Torque teno virus viral load and QuantiFERON Monitor assay and their association with the degree of immunosuppression in kidney transplant patients 

**DOI:** 10.5414/CNP104S02

**Published:** 2025-11-28

**Authors:** Špela Borštnar, Željka Večerić-Haler, Anja Ponikvar Ležaić, Neva Bezeljak, Miha Arnol, Mario Poljak, Maja M. Lunar, Gregor Mlinšek

**Affiliations:** 1 Department of Nephrology, University Medical Center Ljubljana,; 2 Faculty of Medicine, and; 3 Institute of Microbiology and Immunology, Faculty of Medicine, University of Ljubljana, Ljubljana, Slovenia; *These two authors have contributed equally to this work and share first authorship.

**Keywords:** Torque teno virus, QuantiFERON monitor, kidney transplant patients, immunosuppression evaluation

## Abstract

Introduction: Solid organ transplant patients require appropriate immunosuppression to sufficiently control the allorecognition of the graft. Two tests, the QuantiFERON Monitor (QFM) and the Torque teno virus load (TTVL) provide an option to monitor the strength of immunosuppression. Materials and methods: TTVL and QFM were simultaneously determined in kidney transplant patients. Clinical data, microbiological and histopathological findings were collected from the patients’ medical records. Results: 128 TTVL and QFM values were quantified in 107 patients. 69 patients (54%) had recurrent infections in the previous 6 months, 19 (15%) had malignancies, 47 (37%) had a recent kidney biopsy and among them 17 (36%) had histologically proven graft rejection. Results showed that there was no significant correlation between TTVL and QFM (ρ = –0.169, p = 0.061). In patients with histologically proven rejection, TTVL was significantly lower than in patients without rejection (3.64 ± 2.45 vs. 5.02 ± 1.67 log_10_ copies/mL, p = 0.026), but there was no difference between the groups in QFM (1.63 ± 0.67 vs. 1.55 ± 0.80 log_10_ IU/mL, p = 0.735). Patients with known malignancy had lower TTVL compared to patients without it (p = 0.041). No statistically significant difference was observed in TTVL and patients with or without infections (p = 0.278). QFM was not different in patients with or without infection or malignancy. Conclusion: TTVL as an immune marker was associated with transplant rejection. There were no clinically significant associations between QFM and rejection and TTVL or QFM with infections and malignancies. Further prospective studies should be performed to confirm these results.

## Introduction 

Kidney transplantation is a safe and effective treatment for end-stage kidney disease, with immunosuppression playing a vital role in ensuring both patient and graft survival. A major challenge in post-transplant care is balancing immunosuppressive therapy – insufficient suppression increases the risk of rejection, while excessive immunosuppression predisposes patients to infections and malignancies [1, 2]. 

The QuantiFERON Monitor assay (QFM) is an in-vitro diagnostic test that measures the release of interferon-gamma (IFN-γ) in response to stimulation of whole blood with both adaptive and innate immune stimulants. It has potential as a marker of the strength of immunosuppression. However, there are few prospective trials evaluating QFM in kidney transplant patients (KTPs) [3]. 

Torque teno virus (TTV) is a small, non-enveloped, single-stranded DNA virus from the *Anelloviridae* family, with at least 22 known species. Despite its non-pathogenic nature and a prevalence of 80 – 90% in the healthy population, TTV establishes persistent infection and modulates the host immune system. Its replication tends to be higher in immunocompromised individuals, making TTV load (TTVL) a promising functional biomarker of immune status in solid organ transplant (SOT) recipients [4, 5]. Multiple studies have explored the association between TTVL and complications related to over- and under-immunosuppression in KTPs, with varying results. TTVL is influenced by factors such as time post transplant and immunosuppressive regimen but appears unaffected by concurrent antiviral prophylaxis in SOT recipients [2, 3]. 

The aim of this prospective observational study was to analyze the association of TTVL and QFM with rejection, infection, and malignancy in our cohort of KTPs. 

## Materials and methods 

Our cross-sectional clinical study was conducted at the Department of Nephrology, University Medical Center Ljubljana, where the QFM assay is routinely used to assess the cell-mediated immune response as a marker of the strength of immunosuppression in KTPs. In June 2024, the TTVL measurement also became available so that a comparative assessment of these two biomarkers for the strength of immunosuppression was possible. 

This study included 107 KTPs, with a total of 128 TTVL and QFM measurements prospectively collected between July 2024 and February 2025. The attending physician decided to test TTVL and QFM for clinical reasons, emphasizing the need to assess the degree of immunosuppression. 

Clinical data, biochemical parameters, microbiological results, and histopathological findings were extracted from the patients’ medical records. A history of frequent infections was defined as 3 or more infections within the 6 months preceding blood collection. A history of malignancy included either a malignancy treated within the last year or an active malignant disease. A history of graft rejection (borderline, T-cell-mediated, or antibody-mediated) was recorded if it occurred within 2 months before or after blood collection. 

To assess the differences in individuals without immunosuppression, we also conducted a sub-analysis in 22 healthy individuals not receiving immunosuppressive therapy. 

The QFM assay was performed according to the manufacturer’s instructions (Qiagen, Hilden, Germany). Approximately 1 mL of the patient’s blood was collected in a designated test tube and incubated with cellular stimulants targeting Toll-like receptors and T-cell receptors. After incubation, an enzyme immunoassay was used to quantify IFN-γ release, expressed in IU/mL. Based on the manufacturer’s cut-off values, immune status was categorized as follows:

- weak cellular immunity: QFM < 15 IU/mL
- moderate cellular immunity: QFM 15 – 1,000 IU/mL
- strong cellular immunity: QFM > 1,000 IU/mL.

For TTVL measurement, DNA extraction was first performed from 200 µL of plasma using the EZ1&2 Virus Mini Kit v2.0 (Qiagen). TTVL was determined using in-house real-time PCR with primers and probe as previously described [6], amplifying a highly conserved region of 63 base pairs from the 5´UTR. The limit of detection of our TTV method was set at 223 copies/mL with a lower limit of quantification (LLOQ) of 3,000 copies/mL (3.48 log_10_ copies/mL). In samples where the result was < 3,000 copies/mL (< 3.48 log_10_ copies/mL) we considered the number 3,000 copies/mL (3.58 log_10_ copies/mL) for statistical analysis. 

Statistical analysis was performed using GraphPad Prism version 10.4.1. Continuous variables (including TTVL and QFM) were examined for distributional properties, and where appropriate, normalized using log_10_ transformation. Descriptive statistics were expressed as mean ± standard deviation (SD) for normally distributed variables, or as median and interquartile range (IQR) for non-normally distributed data. 

Correlations between continuous variables (e.g., TTVL, QFM, age, time post transplantation) were assessed using Spearman’s rank correlation coefficients. Differences in TTVL and QFM between independent groups (e.g., males vs. females, patients with vs. without rejection, infection, or malignancy) were evaluated using unpaired two-tailed t-tests for normally distributed variables and Mann–Whitney U tests for non-parametric comparisons. Estimation plots were used to visualize group-level mean differences, with 95% confidence intervals (CIs) provided for each contrast. For comparisons with categorical immunological variables (e.g., QFM immune status categories), trend relationships were also explored using correlation analysis. 

Receiver operating characteristic (ROC) curve analysis was used to assess the diagnostic accuracy of TTVL for predicting biopsy-proven rejection. Results are presented as area under the curve (AUC), with corresponding 95% CIs, optimal threshold values, sensitivity, specificity, and positive likelihood ratios (LR^+^). The optimal threshold value for predicting rejection was determined using Youden’s index, which identifies the point on the ROC curve that maximizes the sum of sensitivity and specificity (J = sensitivity + specificity − 1). 

Due to analytical limitations of the TTVL assay, values below the LLOQ of 3,000 copies/mL were considered left censored. For statistical purposes, these values were substituted with 3,000 copies/mL (3.48 log_10_ copies/mL). This approach allowed consistent inclusion of all available samples, but may affect interpretation of medians and interquartile ranges, particularly in subgroups where a high proportion of values fell below the LLOQ (e.g., healthy controls). In these cases, the lower quartile (Q1) could not be precisely estimated, and reported IQRs reflect only the quantifiable portion of the data. 

A p-value < 0.05 was considered statistically significant for all analyses. 

The study received approval from the National Medical Ethics Committee of the Republic of Slovenia (approval number: 0120-216/2019), and all patients provided written informed consent for their voluntary participation. 

## Results 

### Clinical and demographic data 

The comparison group consisted of 22 healthy individuals with no history of chronic diseases, malignancies, or immunosuppressive therapy. This group included 5 males and 17 females, with a mean age of 38 ± 12 years (range: 20 – 61 years). Two participants reported a history of frequent respiratory infections within the past 6 months. 

The KTPs group comprised 107 patients, with a total of 128 simultaneous TTVL and QFM measurements. The clinical and demographic characteristics of these patients at the time of blood sampling are summarized in Table 1. 

### Immunological data in healthy individuals and kidney transplant patients

In the group of 22 healthy individuals, the median QFM was 371.0 IU/mL (IQR 95.73 – 567.0), with values ranging from 4.5 to > 1,000 IU/mL. Most participants (81%) had intermediate cellular immunity, while 5% had low and 5% had strong cellular immunity (data were unavailable for 2 individuals). 

TTVL ranged from undetectable (< 223 copies/mL) to 31,300 copies/mL (2.35 – 4.49 log_10_ copies/mL). In 4 participants (18%), TTVL was undetectable. Since the LLOQ was set at 3,000 copies/mL, 50% of results were at or below this threshold. Accordingly, the median TTVL was 3,000 copies/mL (3.48 log_10_ copies/mL), and values above the quantification limit ranged up to 31,300 copies/mL, resulting in an upper quartile (Q3) of 8,105 copies/mL (3.90 log_10_ copies/mL). As the exact values below the LLOQ were not available, the lower quartile (Q1) could not be precisely determined. 

In KTPs, QFM values ranged from 0.8 to > 1,000 IU/mL, with a median of 81.1 IU/mL (IQR 18.4 – 214.0). Among them, 74.4% had intermediate cellular immunity, 20% had low, and 5.6% had strong cellular immunity. TTVL in KTPs ranged from undetectable < 223 copies/mL to 2.63 ×10^9^ copies/mL (2.35 – 9.42 log_10_ copies/mL), with a median of 10,300 copies/mL (4.04 log_10_ copies/mL) and an IQR of 3,000 – 137,750 copies/mL (3.48 – 5.15 log_10_ copies/mL). Notably, TTVL was undetectable (< 223 copies/mL) in 21 patients (16%), including those receiving triple immunosuppressive therapy. 

**Figure 1:**
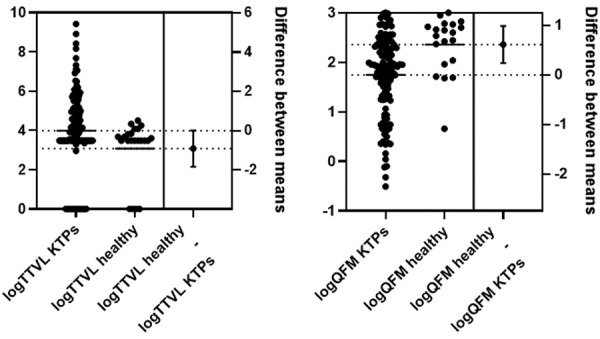
Comparison of Torque teno virus load (TTVL) and QuantiFERON Monitor (QFM) in healthy controls and kidney transplant patients (KTPs), with data normalized using log10 transformation. Log-transformed TTVL values did not differ significantly between healthy individuals and KTPs (unpaired two-tailed t-test, t = 1.958, df = 146, p = 0.052). Log-transformed QFM values were significantly lower in KTPs compared to healthy individuals (unpaired two-tailed t-test, t = 3.254, df = 142, p = 0.001).

When comparing healthy individuals with immunosuppressed KTPs, the healthy group was younger (p < 0.001), had significantly higher QFM values (p = 0.001), and had lower TTVL, with a borderline significant statistical difference (p = 0.052) (Figure 1). 

### Comparison of TTVL and QFM with major clinical parameters in kidney transplant patients 

In KTPs, no significant correlation was found between TTVL and QFM (ρ = –0.169, p = 0.061) or between TTVL and immune status – weak, moderate, strong (ρ = –0.100, p = 0.270). 

There was a significant difference in TTVL between males and females, with males exhibiting higher levels (log TTVL: males 4.52 ± 2.04 vs. females 3.33 ± 1.96 log_10_ copies/mL; p = 0.001). A significant negative correlation was observed between time post transplantation and TTVL (ρ = –0.310, p = 0.002), indicating that viral load decreases over time after kidney transplantation. No significant correlation was found between patient age and TTVL (ρ = –0.140, p = 0.100). Similarly, QFM showed no significant correlations with age (ρ = 0.046, p = 0.614) or time post transplantation (ρ = –0.072, p = 0.428), nor any significant difference between genders (log QFM: males 2.53 ± 0.36 vs. females 2.44 ± 0.43 log_10_ IU/mL; p = 0.194). 

### Comparison of TTVL and QFM in patients with and without frequent infections, malignant disease and rejection 

**Figure 2:**
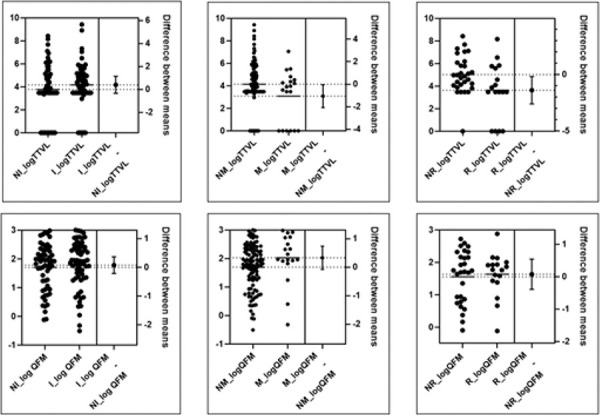
Estimation plots of Torque teno virus load (TTVL) and QuantiFERON Monitor (QFM) in patients with and without frequent infections (left plots), malignant disease (middle plots) and histologically confirmed rejection (right plots) with data normalized using log_10_ transformation. The left panel in each plot displays individual log₁₀TTVL or log₁₀QFM values for each patient in the two groups, with horizontal lines indicating group means. Each dot represents a single patient. The right panel in each plot illustrates the difference of means between groups, with the vertical line indicating the point estimate and the horizontal line representing the 95% confidence interval. NI = no frequent infection group; I = frequent infection group; NM = group without malignant disease; M = group with malignant disease; NR = no rejection group; R = rejection group.

We performed a comparative analysis between patients with and without a history of frequent infections, malignant disease, and histologically confirmed rejection using an unpaired t-test (Table 2) (Figure 2). The differences between groups, along with 95% CIs, are illustrated using estimation plots. There was no difference in TTVL and QFM between patients with or without frequent infections. Regarding malignant disease, there was statistically significant difference in TTVL, but not in QFM. Namely, TTVL values were significantly lower in patients with malignancy compared to those without. The mean difference between groups was –1.06 log_10_ copies/mL (95% CI: –2.08 to –0.05; p = 0.041), indicating that patients with malignancy had, on average, more than a 10-fold lower viral load (Table 2) (Figure 2). 

A significant difference in TTVL was observed between patients with histologically confirmed rejection compared to those without rejection, while no significant difference was found in QFM between these two groups (Table 2) (Figure 2). TTVL was significantly lower in patients who experienced rejection compared to those without rejection. The mean difference between the groups was –1.39 log_10_ copies/mL (95% CI: –2.60 to –0.18; p = 0.026), suggesting that rejection is associated with a markedly lower TTV viral load. Most rejection cases (82.3%) were classified as antibody-mediated rejection (Table 1). Median time between kidney biopsy and TTVL and QFM determination was 0 days (IQR (–22) – 14 days: indicating 22 days prior to biopsy to 14 days after the biopsy). 

The difference between groups and its CI is illustrated in the estimation plot (Figure 2), which displays individual data points, group means, and the estimated mean difference with the 95% CI. 

### ROC analysis of TTVL for predicting rejection after kidney transplantation 

**Figure 3:**
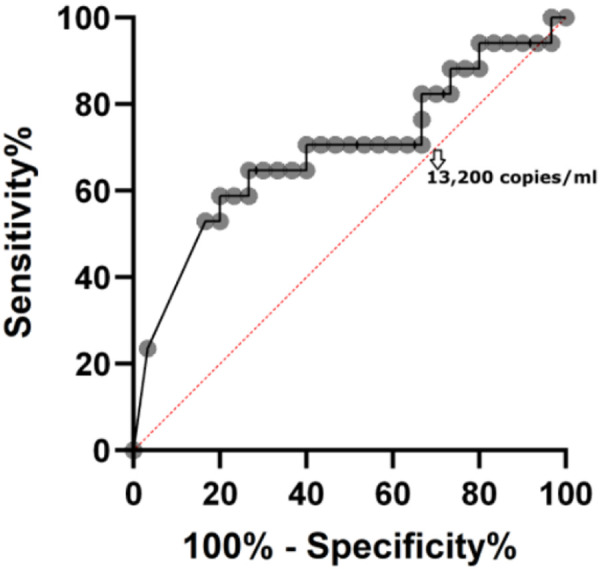
ROC curve evaluating the diagnostic performance of Torque teno virus load for predicting biopsy-proven rejection after kidney transplantation. The area under the curve was 0.690 (95% CI: 0.521 – 0.860; p = 0.032), indicating moderate discriminatory ability. The threshold of < 13,200 copies/mL is indicated on the curve and corresponds to a sensitivity of 64.7% and specificity of 73.3%.

To evaluate the diagnostic potential of TTVL in identifying rejection after kidney transplantation, we performed a ROC curve analysis (Figure 3). The analysis was motivated by the observation of the most significant difference in TTVL between patients with and without biopsy-proven rejection. TTVL was significantly associated with rejection, with an AUC of 0.690 (95% CI: 0.521 – 0.860; p = 0.032), indicating moderate discriminatory ability. At an optimal threshold of < 13,200 copies/mL, the sensitivity was 64.7% and specificity was 73.3%. This suggests that low TTVL values may serve as a useful complementary marker for identifying patients at increased risk of rejection. 

Importantly, very low TTVL values (< 1,500 copies/mL) showed a sensitivity of 23.5% and specificity of 96.7%, resulting in a positive likelihood ratio (LR^+^ = 7.06; 95% CI: 2.84 – 16.17). Despite the low sensitivity, the high specificity supports its potential clinical use in high-suspicion cases where confirming rejection is critical. 

In contrast, TTVL levels below 13,200 copies/mL demonstrated a sensitivity of 64.7% and specificity of 73.3%, with a corresponding LR^+^ of 2.43 (95% CI: 1.86 – 2.91), indicating an approximately 2.4-fold higher likelihood of rejection at this threshold. 

## Discussion 

In our kidney transplant center, therapeutic monitoring of immunosuppressive drugs and assessment of cellular immunity with the QFM assay are two important components of post-transplant evaluation of the strength of immunosuppression. In this study, we investigated TTVL as a novel biomarker for the strength of immunosuppression in KTPs compared to healthy controls. Our results suggest that TTVL may serve as a better indicator of the strength of immunosuppression and was better associated with rejection compared to QFM, while both markers were not associated with recurrent infections. 

As expected, KTPs had lower QFM values and higher TTVL levels compared to healthy individuals, reflecting their immunocompromised state. However, a significant subset of KTPs had unexpectedly low or undetectable TTVL, even in those receiving triple immunosuppressive therapy. This is in contrast to published data, describing TTV prevalence in immunocompromised patients as nearly universal [1] and probably contributes to borderline statistic difference in TTVL among the healthy volunteers and KTPs. This discrepancy may be due to interindividual variability in TTV replication or factors such as the immunosuppressive regimen or the metabolism of immunosuppressive drugs. The proportion of healthy individuals with negative TTVL was consistent with previous literature [4]. 

To our knowledge, this is the first study examining the correlation between TTVL and QFM in adult KTPs. Both markers aim to assess immune competence, with QFM being a relatively established test, though rarely used in KTPs and still insufficiently validated. More recently, attention has shifted to TTVL as a promising biomarker for immune monitoring [3, 4, 7]. Despite both being indicators of immune function, we found no significant correlation between TTVL and QFM. This lack of association could be attributed to their fundamental differences. TTVL likely represents a broader picture of immune status, encompassing both humoral and cellular immunity [8, 9], whereas QFM primarily measures cellular immunity [10]. 

In our cohort, TTVL was significantly associated with gender, with males exhibiting higher TTVL, a finding also observed in previous studies. This may be attributed to the immunomodulatory effects of sex steroid hormones, particularly estrogens, which enhance immune responses in females [11, 12]. Additionally, TTVL decreased with time after transplantation, a trend reported in other studies showing a decline in TTV load 2 – 3 years post transplant [4]. In our patients, the average time after transplantation was 7 years, and a contributing factor could be our center’s practice of gradually reducing immunosuppression in stable patients over time. Surprisingly, no significant correlation could be found between TTVL and age, which contradicts expectations, as aging is typically associated with immunosenescence and higher viral loads. However, conflicting results regarding this association have been reported [4, 13]. 

Unlike previous studies suggesting a strong link between TTVL and infection risk [4], we found no significant association between frequent infections and either TTVL or QFM. However, our results align with some reports that failed to establish this link [2, 3]. One possible explanation is that Margeta et al.’s [3] study found QFM correlated only with bacterial infections, while in our study, we considered both bacterial and viral infections together, potentially obscuring a specific correlation. The absence of an association between TTVL and infections is more challenging to explain. Since the infection history covered the last 6 months before enrolment in the study, it is possible that some patients had already undergone reductions in MMF or MPS in response to previous infections. This common practice in our center, may have influenced our findings. 

In contrast, a significant inverse association was observed between TTVL and malignancy risk, with lower TTVL being associated with a higher probability of malignancy. This is likely due to intentional immunosuppression reduction following cancer diagnosis, leading to improved cellular immunity and lower TTV replication. Namely, all malignancies in our cohort had been diagnosed at least 3 months before blood sampling, ensuring that immunosuppressive adjustments had already been made. Unfortunately, we don’t have data available to confirm this hypothesis. While this association suggests a role for TTVL in tracking immune reconstitution after malignancy, TTVL itself is unlikely to be a direct predictor of cancer development [14, 15]. Furthermore, QFM was not associated with malignancy, a finding that has also been reported in previous studies [16], reinforcing the lack of association between TTVL and QFM. 

A significant association was observed between lower TTVL and a history of rejection, while no such correlation was found with QFM. Patients with histologically confirmed rejection had significantly lower TTVL values than non-rejection patients. ROC curve analysis further supports this observation, with an AUC of 0.69, suggesting moderate discriminatory ability. A threshold of < 13,200 copies/mL yielded a sensitivity of 64.7% and specificity of 73.3%, corresponding to a positive likelihood ratio of 2.4. These results reinforce the potential utility of TTVL as a non-invasive marker of under-immunosuppression and increased risk of rejection in clinical practice. Notably, our findings are in line with previous studies [2, 4], which consistently demonstrated that patients with lower TTVL levels are more likely to experience acute rejection. While TTVL alone may not be sufficient as a stand-alone diagnostic tool, it could serve as a valuable adjunct for immunological monitoring, particularly in identifying patients at higher risk of rejection. 

Conversely, QFM levels did not differ significantly between patients with and without rejection, suggesting that QFM may not reliably reflect immune activation preceding rejection episodes. This may be partly explained by the fact that 82% of rejection cases in our cohort were histologically confirmed antibody-mediated rejections, whereas QFM primarily captures T-cell–mediated immunity. In contrast, TTVL reflects broader immunological suppression, encompassing both innate and adaptive responses [8, 9, 10]. Importantly, the majority of blood sampling was done prior to the biopsy or shortly afterward, ensuring that immune function measurements accurately reflected the rejection status. None of the patients had undergone biopsy more than 2 months before TTVL assessment, and no major changes in immunosuppressive therapy occurred during this period. As TTVL typically reaches a new steady state no earlier than 2 months after immunosuppressive adjustments [17, 18, 19], it is unlikely that our results were influenced by short-term fluctuations in drug levels. 

## Limitations 

The main limitation of our study is the relatively small sample size. However, QFM and TTVL were measured simultaneously under controlled conditions, ensuring a reliable comparison between the two markers. 

When comparing immune markers between the healthy population and KTPs, it is important to consider that the healthy population was younger than the transplant patients, which may have influenced the results. On the other hand, in the case of KTPs, we did not confirm a significant association between TTVL and patient’s age. Further studies with comparable groups need to be conducted in the future. 

While both markers showed potential in assessing the strength of immunosuppression in KTPs, their associations with malignancy, rejection, and infection risk were relatively weak. One challenge in interpreting our results is that infections and malignancies occurred before blood sampling, meaning that our findings may reflect the immune response to these events rather than their actual role in predisposing patients to these complications. This is particularly relevant for malignancies, where reduced immunosuppression following diagnosis could have influenced TTVL and QFM values. Our study was cross-sectional with only a few patients having more than 1 TTVL and QFM values determined within several weeks or months. Future studies should include longitudinal follow-up with serial TTVL and QFM measurements to assess their predictive value more accurately, while also controlling for immunosuppressive adjustments over time. 

## Conclusion 

Our study suggests that TTVL is a more reliable marker than QFM for assessing strength of immunosuppression and is associated with biopsy-proven rejection in KTPs. The association between low TTVL and rejection supports its potential use in guiding immunosuppressive adjustments. However, neither TTVL nor QFM was a strong predictor of recurrent infections, and the association of TTVL with malignancy likely reflects immunosuppressive modifications after diagnosis rather than a causal relationship. Future prospective studies should focus on validating these findings in larger cohorts with longitudinal immune monitoring to refine the role of these biomarkers in clinical practice. 

## Acknowledgment 

The authors would like to thank all of our kidney transplant patients and nurses from the Department of Nephrology for their contribution to our study. 

## Authors’ contributions 

Š.B. and G.M. conceived and designed the study. N.B. and A.P.L. helped with the inclusion of patients. M.M.L. and M.P. performed laboratory determination of TTVL concentrations. Š.B. and G.M. collected all the data. Ž.V.H. performed the statistical analysis. Š.B. and Ž.V.H. wrote the manuscript. Ž.V.H, M.A., M.M.L., M.P., and G.M. read and critically evaluated the manuscript and gave final approval for publication. 

## Funding 

This research was funded by the Slovenian Research and Innovation Agency (research core funding No. P3-0323 and P3-0083). 

## Conflict of interest 

The authors declare no conflict of interest. 

**Table 1.** Clinical and demographic characteristics of kidney transplant patients.

Characteristic	
Age (mean ± SD)	54 ± 14 years (from 21 to 77 years)
Gender (male) (n (%))	61 (57.0%)
Time after transplantation (mean ± SD)	7 ± 6 years (from 6 months to 26 years)
Induction with thymoglobulin, n = 116 (n (%))	11 (9.5%)
Number of immunosuppressive drugs (n (%))	
1	2 (1.6%)
2	78 (60.9%)
3	43 (33.6%)
3 plus daratumumab/eculizumab/rituximab	5 (3.9%)
Calcineurin inhibitor (n (%))	123 (96.1%)
Tacrolimus (n (% – of patients on calcineurin inhibitor))	110 (89.4%)
Cyclosporin (n (% – of patients on calcineurin inhibitor))	13 (10.6%)
Mycophenolic mofetil or sodium (n (%))	85 (66.4%)
Valganciclovir, n = 125 (n (%))	18 (14.4%)
Serum creatinine (mean ± SD)	157 ± 89 µmol/L (from 51 to 503 µmol/L)
Estimated glomerular filtration rate (mean ± SD)	48 ± 22 mL/min/1.73m^2^ (from 80 to 90 mL/min/1.73m^2^)
Frequent infections (n (%))	69 (53.9%)
History of malignant disease (n (%))	19 (14.8%)
History of rejection, percentage of all 107 patients (n (%))	17 (13.2%)
Patients with indication graft biopsy (n (%))*	47 (36.7%)
History of rejection in patients with graft biopsy (n (%))	17 (36.2%)
Antibody-mediated rejection (n (% – of patients with biopsy))	14 (82.3%)
T cell-mediated rejection (n (% – of patients with biopsy))	2 (11.8%)
Mixed or borderline rejection (n (% – of patients with biopsy))	1 (5.9%)

*Only clinically suspicious patients had graft biopsy. Unless otherwise stated, the number of samples was 128.

**Table 2.** Comparative analysis between patients with and without history of frequent infections, with or without malignancy, and with or without histologically confirmed rejection.

	Number of samples (%)	TTVL (copies/mL) (median (IQR), range)	TTVL (log_10_ copies/mL) (mean ± SD)	p-value between TTVL (log_10_ copies/mL)	QFM (IU/mL) (median (IQR), range)	QFM (log_10_ IU/mL)	p-value between QFM (log_10_ IU/mL)
Frequent infections	128 (100%)						
Yes	69 (53.9%)	15,060 (IQR: 3,000 – 139,000), range 0 – 2.63 ×10^9^	4.17±1.87	p = 0.278	81.4 (IQR: 21.65 – 261.5), range 0.31 – 1,000	1.70 ± 1.78	p = 0.588
No	59 (46.1%)	6,240 (IQR: 2,256 – 139,000), range 0 – 2.61 ×10^8^	3.77±2.33		80.8 (IQR: 11.5 – 200), range 0.76 – 955	1.70 ± 0.80	
Malignant disease	128 (100%)						
Yes	19 (14.8%)	8,100 (IQR: 0-34,200), range 0 – 11,300.000	3.09±2.31	p = 0.041*	116.7 (IQR: 77.03-544), range 0.48 – 955.0	2.02 ± 0.87	p = 0.112
No	109 (85.2%)	11,400 (IQR: 3,000 – 144,500), range 0 – 2.63 ×10^9^	4.15±2.02		64.7 (IQR: 17.55 – 200.3), range 0.31 – 1,000	1.69 ± 0.79	
Histologically confirmed rejection	47 (36.7%)						
Yes	17 (36.2%)	3,000 (IQR:1,500 – 477,000), range 0 to 1.45 ×10^8^	3.64 ± 2.45	p = 0.026*	58.9 (IQR: 25.6 – 92.5), range 0.8 – 752.0	1.63 ± 0.67	p = 0.735
No	30 (63.8%)	83,400 (IQR:10,605 – 1.28 ×10^6^), range: 0 to 2.61 ×10^8^	5.02 ± 1.67		51.8 (IQR: 6.6 – 175.5), range 0.8 – 520.0	1.55 ± 0.80	

TTVL = Torque teno virus load; QFM = QuantiFERON Monitor; *statistically significant.
